# Successful bone marrow transplantation in a patient with Omenn syndrome, a rare variant of severe combined immunodeficiency syndrome: A case report

**DOI:** 10.1002/ccr3.9295

**Published:** 2024-08-07

**Authors:** Ubaid Khan, Muhammad Umer, Aiman Muhammad, Arej Iltaf, Abdulqadir J. Nashwan

**Affiliations:** ^1^ Department of Medicine University of Maryland School of Medicine Baltimore Maryland USA; ^2^ Muhammad Medical College Mirpurkhas Pakistan; ^3^ Khyber Girls Medical College Peshawar Pakistan; ^4^ Hamad Medical Corporation Doha Qatar

**Keywords:** bone marrow transplantation, cytomegalovirus, immunodeficiency, Omenn syndrome

## Abstract

**Key Clinical Message:**

Bone marrow transplantation (BMT) saves lives in Omenn syndrome, a severe immunodeficiency disorder. Timely genetic diagnosis and matched donor BMT are crucial. Emphasis on newborn screening and multidisciplinary care improves outcomes for infants with inherited disorders. Prompt intervention and comprehensive management are vital for a successful transplant outcome.

**Abstract:**

Omenn syndrome represents a severe variant of combined immunodeficiency characterized by disregulated immune responses and susceptibility to recurrent infections. We present the case of a 3‐month‐old female infant initially presenting with upper respiratory infection symptoms and a diffuse rash, unresponsive to local treatment. At 4 months of age, the patient underwent allogeneic bone marrow transplantation (BMT) utilizing stem cells from a fully matched sibling donor. Pre‐transplant conditioning included antimicrobial prophylaxis and supportive therapies. Following BMT, the patient received immunosuppressive medications to prevent graft rejection and graft‐versus‐host disease. Clinical monitoring post‐transplant showed timely neutrophil and platelet engraftment, with subsequent follow‐up demonstrating stable clinical parameters and negative cytomegalovirus status. The case highlights the importance of timely diagnosis and treatment in managing severe immunodeficiency disorders, demonstrating the potential for successful outcomes with appropriate timely interventions. Regular monitoring and follow‐up appointments were crucial in ensuring the success of the treatment. This case also emphasizes the significance of multidisciplinary care and genetic testing in identifying and managing rare immunodeficiency disorders. The successful outcome in this case provides hope for improved treatment options and better patient outcomes in the future.

## Graphical abstract

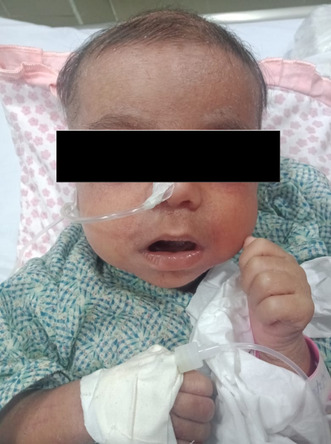

## 1 INTRODUCTION

In 1965, Gilbert S. Omenn first described Omenn syndrome (OS) after he noticed that several individuals in his extended Irish family displayed a wide range of mixed symptoms, varying in severity from person to person. This discovery led to identifying and characterizing OS as a rare genetic disorder affecting the immune system.^1^ OS, a severe form of severe combined immunodeficiency syndrome, is an autosomal recessive syndrome in neonates presenting with erythroderma, lymphadenopathy, hepatosplenomegaly, diarrhea, failure to thrive, high levels of IgE, eosinophilia, and hypogammaglobinemia.^2^ A typical case of OS presents with rash in the first few weeks of life, in addition to skin findings secondary to infiltration of CD4+ helper T cells. An atypical case of OS presents with striking erythroderma and extreme lymphocytosis at birth with skin findings secondary to CD8+ cytotoxic T cells.^3^ OS is predominantly inherited in an autosomal recessive manner, commonly found in marriages between blood relatives.^4^ The syndrome is mostly caused by missense alterations in the genes responsible for recombinase activation “(RAG‐1 and RAG‐2),” which disrupts the process of V(D)J recombination and results in the generation of oligoclonal T lymphocytes.^5^ However, OS may also be caused by alterations in some other genes, including DNA ligase 4, RMRP, CHD7, ILRA7, ILRA2, ADA, ARTEMIS, and the 22q11 microdeletion syndrome. These underlying defects contribute to the development of OS.^5^

OS is a rare fatal syndrome with very high mortality owing to opportunistic infections, and those need to be diagnosed on time.^6^ Due to its rarity, reliable seroprevalence data for OS, specifically, is not readily available. However, the estimated prevalence of this syndrome is roughly 1 in 1,000,000 individuals.^7^ Skin biopsy and genetic testing are helpful investigations and should be performed early for the ultimate diagnosis.^8^ OS should also be considered a part of the differential diagnosis when prenatal ultrasound shows altered findings in the tegument system.^9^ The gold standard treatment for OS is hematopoietic stem cell transplantation (HSCT),^10^ and despite being a potential treatment option, HSCT has only a moderate prognosis for individuals with OS.^11^ A study showed that mortality rate even after treatment with HCST is 48% which is quite high.^12^ This is primarily due to complications caused by the highly activated Omenn T cells, which can delay the process of T‐cell engraftment and result in a high graft failure rate.

## 2 CASE REPORT

### 2.1 Presenting complain

A 3‐month‐old female infant was presented at a local health facility with upper respiratory infection symptoms, including nasal congestion and widespread body rash. Local treatment was initiated, but the patient did not show any signs of improvement. Due to the persistence of signs and symptoms, the patient was transferred to a tertiary care hospital for further evaluation.

### 2.2 Physical examination

**FIGURE 1:**
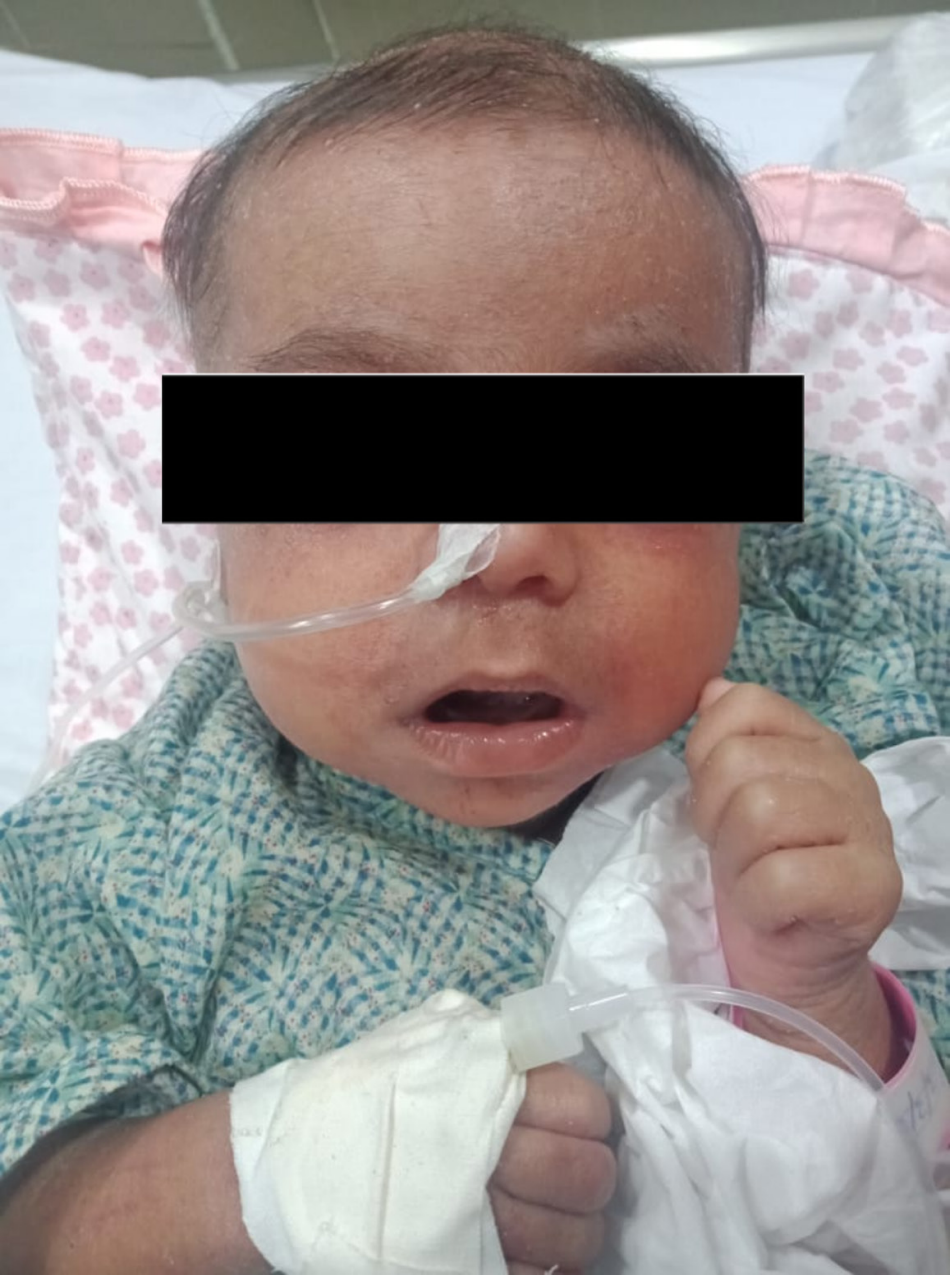
Image showing the diffuse scaling.

Physical examination revealed a lethargic patient with diffuse thick scaling starting from the vertex of the scalp with progressive involvement of the forehead with some pustules on his face and trunk had been noted since birth (Figure 1), associated with alopecia, lymphadenopathy, and hepatomegaly.

### 2.3 Laboratory findings

Laboratory findings revealed a leukocyte count lower than the normal range with marked eosinophilia, a very prominent complete blood count (CBC) finding. A lymphocyte immunophenotyping test was conducted, and a significant decrease in the absolute count of CD3+ total T and CD19+ total T lymphocytes was observed. A decline in both subsets of T lymphocytes (004+ and 008+) and an elevation in the population of CD56+ natural killer cells was also observed.

Blood electrolytes, including serum sodium, potassium, chloride, and bicarbonate levels, indicated that all values were within the normal range. Similarly, CBC such as the red blood cells, mean corpuscular volume, mean corpuscular hemoglobin (Hb), mean corpuscular Hb concentration, red blood cell distribution width, white blood cells, neutrophils, lymphocytes, basophils, and platelets exhibited normal values. Nevertheless, abnormalities were observed in eosinophils and monocytes. Additionally, after 5 days, the blood culture was conducted, which yielded no evidence of microbial growth.

After a month, sequence analysis and deletion/duplication testing of the 130 genes listed in the genes analyzed section were performed. Two pathogenic variants were identified in RAG2, which is associated with autosomal recessive SCID. One likely pathogenic variant and one variant of uncertain significance were identified in DOCK8. Based on genomic sequencing and abnormalities in leukocyte count, this case was diagnosed as OS. These mutations disrupt lymphocyte development, leaving the patient vulnerable to infections.

Later, at the age of 4 months, the patient with a known diagnosis of SCID was admitted to the specialty hospital with the primary complaint of requiring an allogenic bone marrow transplant (BMT).

Several baseline laboratory investigations were conducted on admission to assess her overall health status. The patient's CBC revealed a Hb level of 10.3 g/dL, an elevated white blood cell counts of 20.92/μL, and a platelet count of 253 × 10^3^/μL. Renal function tests showed urea levels of 13 mg/dL and a creatinine level of 0.3 mg/dL. Electrolyte levels were relatively stable, with sodium 139 mmol/L and calcium 10.4 mmol/L. Liver function tests indicated a total bilirubin level of 0.3 mg/dL and an alanine aminotransferase level of 34 U/L. Cytomegalovirus (CMV) status was negative as of the last assessment, a week after the diagnosis of OS. Echocardiography and chest X‐ray showed no significant findings. However, there was hepatosplenomegaly on abdominal ultrasound. The patient's serology tested negative for hepatitis B surface antigen, anti‐hepatitis C virus, human immunodeficiency virus, and syphilis. Her C‐reactive protein was notably elevated at 157 mg/L, indicating an ongoing inflammatory process, and the patient tested negative for COVID‐19.

### 2.4 Treatment

The course of treatment began with preconditioning, which involved the administration of antifungal, antiviral, anti‐helminthic, oral antibiotic, and mouthwash medications. A Hickman line was inserted to facilitate treatment. After a month, conditioning for the BMT commenced, incorporating fludarabine and treosulfan, along with supportive therapies such as acyclovir and benzydamine HCl. chlorhexidine mouthwash, and nystatin oral drops. Granulocyte colony‐stimulating factor was administered to stimulate white blood cell production.

At the onset of her hospitalization, cultures were found to be positive, indicating the presence of underlying infections, which were treated before HSCT. However, CMV was not detected, and cyclosporine levels were reported weekly. Blood products were transfused as needed during the stay. The patient also received a comprehensive pre‐conditioning regimen, including antibiotics such as meropenem, linezolid, clarithromycin, and teicoplanin, as well as antifungal medication. This preparatory phase aimed to optimize her condition for the subsequent BMT. The following medications were administered during the conditioning phase, including fludarabine and treosulfan.

Finally, at the age of 5 months, the patient underwent a peripheral stem cell transplantation, and the procedure utilized stem cells from a perfectly matched 6/6 HLA donor, who happened to be the patient's sister. She was also given immunosuppressive medications, including cyclosporine and mycophenolate mofetil (MMF), to prevent graft rejection and graft‐versus‐host disease. Regular monitoring included assessing CMV and cyclosporine levels weekly to track the patient's response to treatment and make necessary adjustments. She also received IVIG therapy twice to provide passive immunity and protect against infections in immunocompromised individuals. On the 10th Day post‐transplant, she achieved neutrophil engraftment, marking a critical milestone in her recovery. Platelet engraftment followed on the 14th Day post‐transplant, indicating the successful integration of the transplanted bone marrow.

Upon discharge, the results of investigations revealed the following: Hb: 7.6 g/dL, normal white blood cell count: 6.04 × 10^3^/μL, absolute neutrophil count (ANC): 2.09 and platelet count: 57 × 10^3^/μL (Table 1). Renal function tests (RFTs) indicated stable values with urea at 13 mg/dL and creatinine at 0.2 mg/dL. Electrolyte levels were within the normal range, with sodium 137 mmol/L, potassium 4.7 mmol/L, and calcium 8.4 mmol/L. Liver function tests (LFTs) showed a total bilirubin level of 0.4 mg/dL and an ALT level of 21 U/L. Her CMV status remained negative throughout the post‐op period.

**TABLE 1:** Post‐transplantation laboratory parameters.

Laboratory parameter	Patient	Normal range
WBC, cells/mm^3^	5.1 × 10E9/L	5–15
Lymphocytes, %, cells/mm^3^	24.3	67–80
Neutrophils, %, cells/mm^3^	30.2	20–33
Eosinophils, %, cells/mm^3^	29.6	2–6
Platelets, ×10^3^ cells/mm^3^	334 × 10E9/L	210–650
Hemoglobin, g/dL	11.4	9.4–13
T‐cell CD3+, %, cells/mm^3^	1487	1900–5900
NK CD16+, CD56+ %, cells/mm^3^	312	160–950
B‐cell CD19+, %, cells/mm^3^	692	610–2500
IgG, mg/dL	9.9	2.03–9.34 g/L
IgM, mg/dL	1.25	0.17–1.5 g/L
IgA, mg/dL	0.56	0.08–0.91 g/L
CMV	Negative	Negative

### 2.5 Medications and follow‐up

The patient underwent HSCT at the age of 5 months. Upon discharge, the treatment plan for the patient at home included several medications and supportive measures: Fluconazole tablet once daily, MMF syrup 2 mL twice daily, cyclosporine syrup 0.15 mL daily, acyclovir syrup 1 mL three times daily, benzydamine HCl. chlorhexidine mouthwash every 6 h, nystatin drops 2.5 mL used every 6 h, vitamin B12 drops once daily, and vitamin D drops once daily.

Follow‐up appointments scheduled every third day for 2 weeks, dressing changes and heparinization of the Hickman line, clinical examination to monitor height, weight, and complications, immediate reporting in case of any events, such as fever, rash, bleeding, seizures, etc., ongoing investigations during follow‐up, including CBC, serum electrolytes, RFTs, LFTs, blood sugar levels, urine analysis, cyclosporine level and CMV DNA quantitative analysis; evaluation of donor chimerism on Day 30+ post‐transplant to assess the success of the BMT.

During her follow‐up visits, she presented with a favorable clinical profile and reported no fever, petechial, or bruise and no complaints of diarrhea or vomiting. Furthermore, she appeared to be an active, alert, and playful child, demonstrating an encouraging state of health. Her vital signs remained within normal ranges, with her pulse at 132 beats per minute, a temperature of 98°F, a respiratory rate of 30 breaths per minute, and an oxygen saturation (SPO_2_) level of 99%.

Investigations indicated that routine laboratory parameters were within acceptable range. Importantly, her CMV status was negative, a significant finding for an immunocompromised patient. Additionally, cyclosporine levels, a critical marker for monitoring the success of her BMT, were measured on two different occasions and were within acceptable range.

Her follow‐up plan consisted of weekly visits to monitor her progress and ensure the continued success of her BMT. Weekly follow‐ups include dressing changes, hub heparinization, clinical examinations for height and weight, and meticulous assessment for complications. Any untoward events like fever, rash, bleeding, fits, altered consciousness, headache, or vomiting must be promptly reported to the bone marrow transplant team. Investigations on follow‐up encompass CBC, peripheral blood smear, RFTs, BSR, urine dipstick/reagent, and CMV DNA to monitor the patient's post‐transplant progress and promptly address any arising issues.

Despite the successful BMT, this case highlighted some significant challenges associated with OS. Although the patient was fortunate to have a perfectly matched sibling donor, relying solely on this possibility can be a hurdle. Early detection through newborn screening programs could significantly improve outcomes by allowing for prompt intervention and potentially a wider range of donor options.

## 3 DISCUSSION

This case report describes the treatment journey of a 3‐month‐old female infant who suffered from SCID and underwent a BMT from her HLA‐matched sister.

The history of OS can be traced back to 1965.^12^ That time, Omenn published a detailed account of a particular OS case. The case involved an Irish–American family and exhibited common characteristics of the syndrome, such as recurring infections, skin eruptions, eosinophilia, and various respiratory and gastrointestinal symptoms.^13^ Additionally, the affected individuals experienced failure to thrive. In OS, the count of T lymphocytes can be either normal or high, while there is a slight increase in eosinophils.^1^, ^8^ In our case, the patient also suffered from upper respiratory infection, and a decline in both subsets of T‐Lymphocytes (004+ and 008+) was also observed.

In 1998, researchers discovered hypomorphic RAG alterations were the main reason behind OS.^14^ In humans, when there is only a partial RAG function, it results in a condition called OS.^6^ On the other hand, if the RAG gene is completely inactivated, it leads to the T‐B‐SCID phenotype.^15^ In our case, both lymphocytes were found to be decreased.

In our case, identifying pathogenic mutations in RAG2 genes confirmed OS and explained the patient's compromised immune system. This genetic fingerprint directly informed the BMT approach, offering a targeted treatment with a higher chance of success compared to broader immune therapies. While the likely pathogenic variant in DOCK8 might have contributed to the condition, its full impact remains unclear, highlighting the need for further research on DOCK8 variants in OS for future advancements in treatment strategies.

Based on prior research, HSCT has emerged as a key therapeutic strategy for congenital immunological disorders, owing to the crucial role played by myeloid cells in the functioning of the immune system.^1^, ^16^ Our patient was diagnosed at the age of 3 months, followed by the transplantation procedure at the age of 5 months. The age at which a patient undergoes HSCT is a significant variable that can potentially impact the overall success of the procedure.^4^ Several studies have demonstrated that administering early HSCT in neonates can enhance thymic output and improved survival rates among patients with SCID.^17^ However, contrasting findings have suggested that the clinical condition of patients with OS at the time of HSCT is a more reliable predictor of successful outcomes than their age.^18^

Additionally, our patient was found to have a negative result for CMV during the time of transplantation and post‐op period. However, the study conducted by Shamsian et al. reported that the donor's CMV status was not specified.^19^ Before the transplantation procedure, the individual exhibited a positive result on the CMV test. The impact of CMV infection on individuals with SCID is well‐documented since it can lead to severe complications and even mortality.^20^ The management of infection following HSCT is challenging due to the constraints posed by toxicities associated with antiviral drugs and the potential emergence of drug‐resistant strains.

The success rates of HSCT are significantly greater in individuals who undergo the transplant before the emergence of infection and at an early stage of life, namely at 3.5 months of age or younger.^21^ Additionally, the donor status and the procedures used to condition the patients before transplantation are crucial factors that influence the procedure's success. Unless a stem cell transplant is administered, all instances of OS have resulted in fatal results within a period of 2 months following delivery. Furthermore, it is commonly seen that these individuals often have a delayed diagnosis, resulting in a heightened susceptibility to mortality as a consequence of unaddressed subsequent consequences.^13^ Therefore, it is imperative to implement newborn screening to optimize the ultimate result.

## AUTHOR CONTRIBUTIONS

**Ubaid Khan:** Writing – original draft; writing – review and editing. **Muhammad Umer:** Writing – original draft; writing – review and editing. **Aiman Muhammad:** Writing – original draft; writing – review and editing. **Arej Iltaf:** Writing – original draft; writing – review and editing. **Abdulqadir J. Nashwan:** Writing – original draft; writing – review and editing.

## FUNDING INFORMATION

This study received no funding.

## CONFLICT OF INTEREST STATEMENT

The authors declare no conflicts of interest.

## ETHICS STATEMENT

Ethical approval was not required for this study in accordance with local or national guidelines.

## CONSENT

Written informed consent was obtained from the patient to publish this report in accordance with the journal's patient consent policy.

## Data Availability

All data generated or analyzed in this study are included in this published article. Further inquiries can be directed to the corresponding authors.
